# Different vasodilatation characteristics among the main cerebral arteries in fetuses with congenital heart defects

**DOI:** 10.1038/s41598-018-22663-5

**Published:** 2018-03-14

**Authors:** Qinghai Peng, Shi Zeng, Qichang Zhou, Wen Deng, Tao Wang, Ya Tan, Yushan Liu

**Affiliations:** 1 Department of Ultrasound Diagnosis, the Second Xiangya Hospital, Central South University, Changsha, Hunan 410011 China; 2 Department of Obstetrics and Gynecology, the Second Xiangya Hospital, Central South University, Changsha, China; 3 Department of Pediatrics, the Second Xiangya Hospital, Central South University, Changsha, China

## Abstract

To observe Doppler changes in the three main cerebral arteries in fetuses with congenital heart defects (CHDs). The pulsatility index (PI) values of the anterior cerebral artery (ACA), middle cerebral artery (MCA), and posterior cerebral artery (PCA) were prospectively compared in 78 CHD fetuses and 78 normal control fetuses. Correlations between the cerebral artery PIs and the neurodevelopment scores (psychomotor development index [PDI] and mental development index [MDI]) were assessed. The MCA-PI was decreased significantly in fetuses with hypoplastic left heart syndrome (HLHS). The ACA-PI was reduced significantly in fetuses with HLHS, fetuses with left-sided obstructive lesions (LSOLs) and fetuses with transposition of the great arteries. The PCA-PI was significantly smaller in fetuses with HLHS and fetuses with LSOLs. More fetuses presented signs of cerebral vasodilatation of the ACA than the MCA for certain types of CHD (P < 0.05). The ACA-PI was positively correlated with the PDI and MDI scores in fetuses with CHDs (r^2^ = 0.26, 0.20, P < 0.01). The MCA-PI was only positively correlated with the PDI scores (r^2^ = 0.15, P < 0.01). The ACA exhibited signs of vasodilatation more frequently and severely than the MCA. The ACA-PI appears to be more sensitive for predicting abnormal neurodevelopmental outcomes than the MCA-PI.

## Introduction

Emerging studies have demonstrated that fetuses with congenital heart defects (CHDs) are at an increased risk of brain abnormalities and/or neurodevelopmental (ND) delays in the absence of known major aneuploidy or genetic disorders. Although the underlying mechanisms are not fully understood, aberrant fetal cerebral hemodynamics may be an important risk factor. Our previous studies and others have shown changes in the fetal cerebral circulation in fetuses with CHDs. Such changes include a lower middle cerebral artery pulsatility index (MCI-PI)^1–8^, a lower cerebral placental ratio (CPR = MCA-PI/UA-PI), where UA represents the umbilical artery^1–3,7,8^, and increased cerebral perfusion^1,5^. These changes indicate brain-sparing in CHD fetuses and result in relatively increased blood flow to the brain.

The MCA, the most commonly studied cerebral vessel, is considered the clinical standard for hemodynamically evaluating the fetal brain. However, its applications are challenged by regional hemodynamic variations in the brain circulation. Several studies have demonstrated the existence of regional brain redistribution processes. Both the anterior cerebral artery (ACA)^9,10^ and the posterior cerebral artery (PCA)^11^ present earlier signs of vasodilatation than the MCA in fetuses with intrauterine growth restriction (IUGR). Dubiel M. *et al*.^12^ also found that the ACA-PI had a greater correlation with adverse perinatal outcomes than the MCA in pregnancies complicated by pregnancy-induced hypertension. However, the Doppler waveforms of such arterial territories, the ACA and PCA, have not been studied in fetuses with CHDs.

Thus, the purpose of this study was to observe the Doppler changes in the three main cerebral arteries in CHD fetuses and evaluate the relationship between signs of brain-sparing in each artery and neonatal ND test results.

## Results

### General condition

In all, 175 fetuses were enrolled during the study period, but 13 and 6 fetuses were excluded due to excessive fetal motion and loss of follow-up, respectively. A total of 156 fetuses were finally included: 17 fetuses with HLHS, 19 fetuses with LSOLs, 22 fetuses with RSOLs, 20 fetuses with TGA, and 78 gestational age-matched normal fetuses. The normal control group consisted of equal number and similar GA of each CHD subgroup. All prenatal diagnoses of CHD were in agreement with the postnatal echocardiograms or autopsy findings. Table 1 lists the clinical demographics of all the enrolled fetuses. The gestational age and EFW at the first examination were not significantly different between the CHD and normal control groups. The birth weight of CHD fetuses was smaller than that of the controls. At the end of pregnancies, 6 CHD fetuses were small for gestational age at birth or late-onset intrauterine growth restricted, including three with HLHS, one with LSOLs and two with RSOLs. There was no significant difference in the UA-PI between the two groups.

**Table 1:** Clinical demographics of the enrolled fetuses (n = 156).

Variable	Fetuses with CHDs (n = 78)	Controls (n = 78)	P value
Maternal age (years)	26.6 ± 5.4	26.6 ± 4.8	0.97
Nulliparous, n (%)	42 (54%)	50 (64%)	0.25
AR pregnancy	16 (21%)	10 (13%)	0.28
Family history of CHDs	7 (9%)	5 (6%)	0.77
GA at diagnosis (weeks)	24.1 ± 3.1	24.1 ± 3.1	0.98
EFW at diagnosis (g)	712.3 ± 351.8	719.8 ± 404.1	0.90
UA-PI	1.07 ± 0.25	1.06 ± 0.18	0.65
GA at birth (weeks)	38.7 ± 2.0	39.3 ± 1.1	0.03
Birth weight (g)	3233.9 ± 496. 2	3473.3 ± 261.0	0.000
Birth weight centile	28 ± 19	51 ± 6	0.000
CHD subgroups (n, %)			
HLHS	17 (21.8%)		
LSOLs	19 (24.4%)		
Aorta hypoplasia/coarctation	9 (11.5%)		
Aortic stenosis	3 (3.8%)		
Interrupted aortic arch	7 (9%)		
RSOLs	22 (28.2%)		
TOF	12 (15.4%)		
pulmonary stenosis	5 (6.4%)		
PA-IVS	2 (2.6%)		
Ebstein’s anomaly	1 (1.3%)		
Tricuspid atresia	2 (2.6%)		
TGA	20 (25.6%)		

AR, assisted reproduction; CHD, congenital heart defect; GA, gestation age; EFW, estimated fetal weight; HLHS, hypoplastic left heart syndrome; LSOLs, left-sided obstructive lesions; RSOLs, right-sided obstructive lesions; TOF, tetralogy of Fallot; PA-IVS, pulmonary atresia with intact ventricular septum; TGA, transposition of the great arteries; UA-PI, umbilical artery pulsatility index.

The clinical and postnatal information of CHD fetuses are listed in Table 2. There were 65 live births. 48 cases underwent cardiac surgery at a mean age of 53 ± 45 days. Five infants dies during the peri-surgical period (within 30 days of surgery), including three infants with HLHS, one with TOF and one with tricuspid atresia. 32 cases had post-surgical complications, most including low cardiac output syndrome (31%), hyoxemia (25%) and infection (25%). In total, 38 infants with CHD underwent ECMO, including 4 cases pre-operatively, 28 cases intra-operatively, and 6 cases post-operatively.

**Table 2:** Clinical and postnatal outcomes of the CHD fetuses (n = 78).

Characteristics	Fetus with CHD (n = 78)
Perinatal outcome	
IUFD, n (%)	4(5%)
TOP, n (%)	9(11.5%)
Alive birth, n (%)	65(83.3%)
Postnatal treatment and outcome	
Follow-up period (months)	32 ± 5
Before-surgery death, n (%)	2(2.5%)
Peri-surgery death, n (%)	5(6%)
cardiac surgery, n(%)	48(61.5%)
at age (days)	53 ± 45
duration (hours)	4.7 ± 1.6
ECMO n	38
duration (days)	5 ± 1
Post-surgery complications,n(%)	32(66.7)
LCOS	10
hyoxemia	8
infection	8
arrthymia	3
MODS	2
Respiratory failure	1

IUFD, intrauterine fetal death; TOP, termination of pregnancy; ECMO:extracorporeal membrane oxygenation; LCOS, low cardiac output syndrome; MODS, multiple organ disfunction syndrome.

### Cerebral arteries PIs

Doppler data were acquired for all three cerebral arteries in all fetuses. The head biometry z scores and cerebral circulation Doppler values are shown in Table 2. There were no differences in head biometry between the CHD and control groups. The biparietal diameter (BPD) and head circumference (HC) z scores in CHD fetuses were in the normal range (-2 ~ +2). Overall, CHD fetuses showed a significant reduction in the PIs of the cerebral arteries compared with the controls. However, different patterns were observed in each vessel: the MCA-PI was significantly decreased in fetuses with HLHS. The ACA-PI was significantly reduced in fetuses with HLHS, fetuses with LSOLs and fetuses with TGA. The PCA-PI was significantly smaller in fetuses with HLHS and fetuses with LSOLs (Table 3).

**Table 3:** Head biometrics and cerebral circulation in the cohort (n = 156).

	Normal (n = 78)	HLHS (n = 17)	P	LSOLs (n = 19)	P	RSOLs (n = 22)	P	TGA (n = 20)	P
BPD z score	0.02 ± 0.72	−0.52 ± 0.96	0.23	−0.46 ± 0.98	0.3	−0.20 ± 1.02	0.88	0.17 ± 0.88	0.91
HC z score	0.08 ± 0.82	−0.67 ± 0.92	0.14	−0.62 ± 0.95	0.18	−0.44 ± 1.0	0.55	0.14 ± 0.99	0.89
ACA-PI	1.83 ± 0.21	1.01 ± 0.18	<0.001	1.41 ± 0.37	<0.01	1.80 ± 0.28	0.96	1.55 ± 0.29	<0.01
MCA-PI	1.94 ± 0.19	1.30 ± 0.24	<0.001	1.74 ± 0.29	0.07	1.90 ± 0.15	0.76	1.86 ± 0.18	0.38
PCA-PI	1.64 ± 0.19	1.10 ± 0.25	<0.001	1.31 ± 0.43	0.03	1.63 ± 0.26	0.92	1.46 ± 0.30	0.06

P, compared with the normal control group analyzed by ANOVA with post hoc Games-Howell testing.

BPD, biparietal diameter; HC, head circumference; ACA, anterior cerebral artery; MCA, middle cerebral artery; PCA, posterior cerebral artery; PI, pulsatility index; HLHS, hypoplastic left heart syndrome; LSOLs, left-sided obstructive lesions; RSOLs, right-sided obstructive lesions; TGA, transposition of the great arteries.

**Figure 1:**
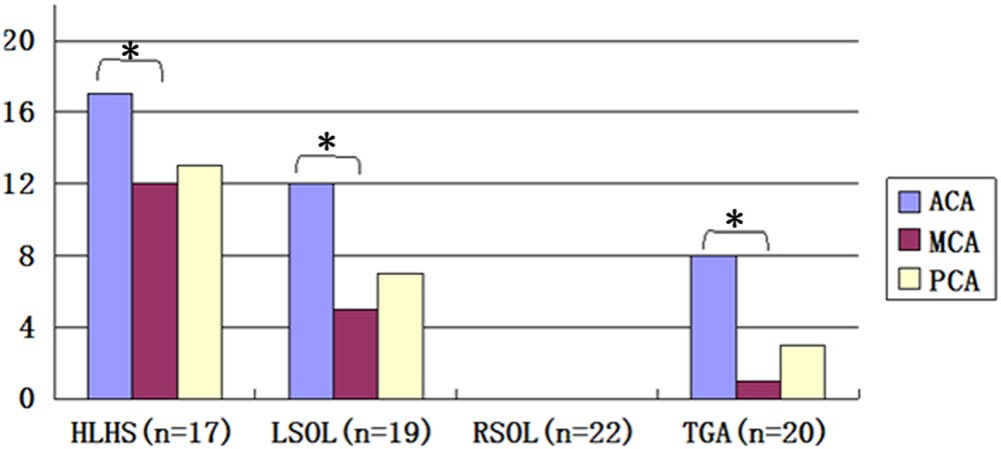
The number of cases with signs of cerebral vasodilatation in the CHD fetus subgroups. Many more fetuses presented signs of cerebral vasodilatation of the ACA than the MCA. *P < 0.05. ACA, anterior cerebral artery; MCA, middle cerebral artery; PCA, posterior cerebral artery; HLHS, hypoplastic left heart syndrome; LSOLs, left-sided obstructive lesions; RSOLs, right-sided obstructive lesions; TGA, transposition of the great arteries.

For certain types of CHDs, more fetuses presented signs of cerebral vasodilatation of the ACA than the MCA (P < 0.05, Fig. 1). In fetuses with HLHS, signs of cerebral vasodilatation of the ACA, MCA and PCA were present in 17 cases (100%), 12 cases (70.6%) and 13 cases (76.5%), respectively. In fetuses with LSOLs, signs of cerebral vasodilatation of the ACA, MCA and PCA were present in 12 cases (63.2%), 5 cases (26.3%) and 7 cases (36.8%), respectively. In fetuses with TGA, signs of cerebral vasodilatation of the ACA, MCA and PCA were present in 8 cases (40%), 1 case (5%) and 3 cases (15%), respectively.

### Neurodevelopment Testing

**Figure 2:**
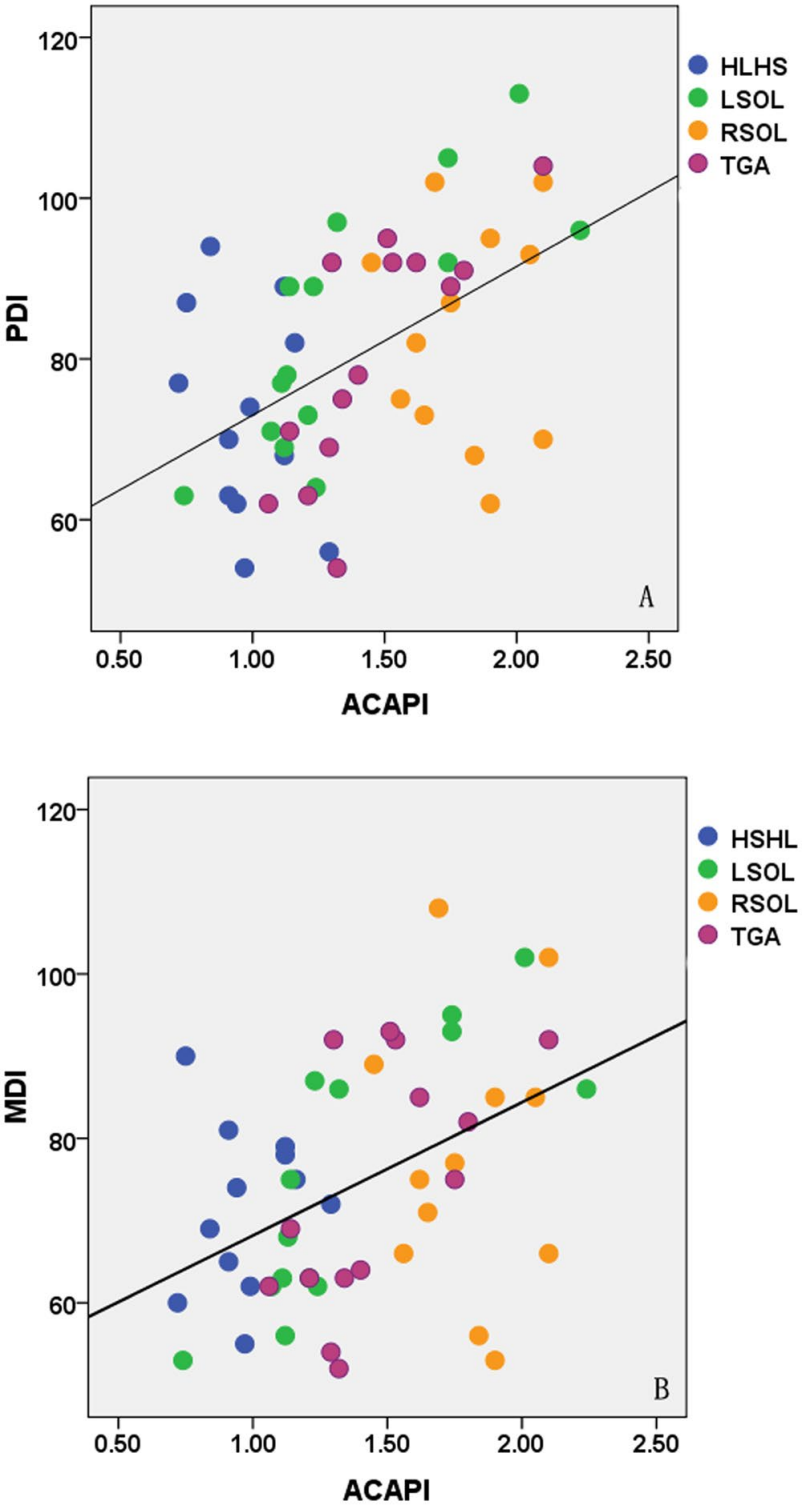
The relationship between the ACA-PI and ND test scores at 6 months in fetuses with CHDs. The ACA-PI was positively correlated with the PDI (**A**) and MDI scores (**B**). ACA, anterior cerebral artery; PI, pulsatility index; PDI, psychomotor development index; MDI, mental development index.

**Figure 3:**
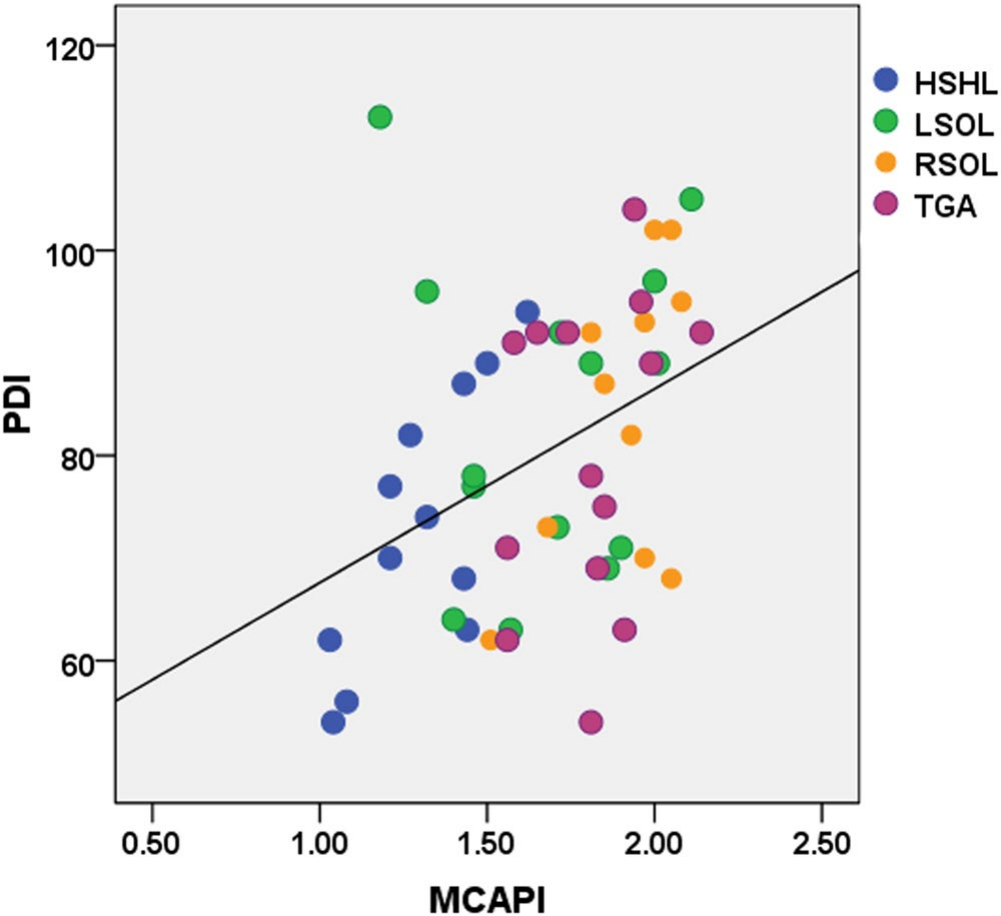
The relationship between the MCA-PI and ND test scores at 6 months in fetuses with CHDs. The MCA-PI was positively correlated with the PDI scores. MCA, middle cerebral artery; PI, pulsatility index; PDI, psychomotor development index.

In total, 91 fetuses underwent ND testing at a mean age of 6.2 ± 0.1 months, including 12 fetuses with HLHS, 14 with LSOLs, 12 with RSOLs, 14 with TGA and 39 normal controls. Additionally 23 CHD infants had surgery and/or underwent ECMO prior the ND testing. The PDI and MDI scores of the CHD group were significantly lower than those of the control group (80 ± 14 vs 103 ± 8 and 75 ± 15 vs 99 ± 7, respectively, P < 0.05). As for CHD fetuses, the ACA-PI was positively correlated with the PDI and MDI scores (r^2^ = 0.26, 0.20, P < 0.01) (Fig. 2), the MCA-PI was only positively correlated with the PDI scores (r^2^ = 0.15, P < 0.01) (Fig. 3) and there were no significant correlations between the PCA-PI and ND test scores. As for normal controls, there were no obvious correlations between PI of all the cerebral arteries and neurodevelopmental outcome.

The intra- and interclass correlation coefficients for the ACA-PI were 0.95 (95% CI, 0.91–0.97) and 0.89 (95% CI, 0.78–0.94), while those for the PCA-PI were 0.94 (95% CI, 0.86–0.99) and 0.88 (95% CI, 0.74–0.95).

## Discussion

To the best of our knowledge, this is the first study to evaluate ACA and PCA Doppler changes and demonstrate different vasodilatation characteristics among the three main cerebral arteries in CHD fetuses.

The MCA was the first and is traditionally the most commonly studied brain vessel in fetuses. Previous studies assessing MCA Doppler data in fetuses with CHDs have reported conflicting conclusions: many studies have reported a significantly decreased MCA-PI in fetuses with CHDs^1–8^, while several studies have stated there was no significant difference in the MCA-PI or the MCA resistance index between CHD and control fetuses^13–15^. Moreover, altered Doppler indices, such as the MCA-PI, MCA-RI and CPR, were detected in almost all fetuses with HLHS. Although other forms of CHDs also lead to decreased brain flow and/or oxygen perfusion (e.g., TGA and LSOLs), few studies have reported decreased MCA-PI values in fetuses with TGA^16^ or aortic coarctation^8^. Insufficient sensitivity and/or late onset vasodilatation of the MCA may be one explanation for such contradictions.

Our study showed that the MCA-PI was decreased only in fetuses with HLHS, while the ACA-PI was reduced in fetuses with HLHS, LSOLs or TGA, and the PCA-PI was reduced in fetuses with HLHS or LSOLs, indicating the presence of regional variations of brain perfusion in fetuses with CHDs. The ACA originates at the internal carotid and travels at a right angle with penetrating branches supplying blood to most of the superior-medial parietal lobes and portions of the frontal lobes. The MCA, as the terminal branch of the internal carotid artery, travels laterally and supplies the lateral frontal, temporal and parietal lobes. The PCA, the terminal branch of the basilar artery, supplies the occipital lobes and inferior temporal lobes^17^. Therefore, the MCA alone may be insufficient for demonstrating cerebral hemodynamics in other vascular territories. We intentionally performed Doppler measurements in cerebral artery segments prior to the anterior/posterior communicating arteries in this study to reduce interference from communicating arteries in the circle of Willis. In our previous study^5^, we showed that ACA power Doppler indices, such as the vascularization index, flow index, and vascularization flow index, were greater in fetuses with HLHS, LSOLs or TGA, and we demonstrated that cerebral blood perfusion was increased in the ACA territory in most CHD fetuses. This phenomenon is consistent with the results of this study, indicating that the redistribution favors the frontal lobes instead of the lateral and posterior parts of the brain in CHD fetuses.

Our study found that in any CHD fetus subgroup, more fetuses exhibited an abnormal PI for the ACA than the MCA, indicating that the ACA-PI may be more sensitive to vascular impedance than the MCA-PI in fetuses with CHDs. Other studies on fetuses of other high-risk pregnancies also reported that the ACA showed signs of vasodilatation more frequently and earlier than the MCA. Dubiel M. *et al*.^12^ demonstrated that Doppler signs of brain-sparing in the ACA, MCA, and PCA were found in 90, 52, and 65 fetuses suspected of having chronic hypoxia, respectively. Cruz-Martinez R. *et al*.^18^ found that the proportion of fetuses with an abnormal ACA-PI was higher than that of fetuses with an abnormal MCA-PI in the context of small for gestational age (17.2% vs 10.8% at 37 weeks of gestation and 20.9% vs 16.4% before delivery). Moreover, an abnormal ACA-PI occurs an average of one week earlier than an abnormal MCA-PI. Figueroa-Diesel *et al*.^10^ observed that some fetuses with IUGR in hemodynamic stage 1 showed a significantly reduced ACA-PI, while no fetuses had abnormal MCA-PIs at this stage. Benavides-Serralde *et al*.^9^ explored the MCA and two segments of the ACA in IUGR fetuses and observed that the first segment of the ACA exhibited a reduced PI earlier than the MCA. In terms of PCA-PI, other teams^10,11^ have reported that IUGR fetuses showed vasodilatation in the PCA earlier than in the MCA regardless of the UA-PI value. However, our study showed that there was no difference between MCA and PCA vasodilatation in the CHD fetuses.

Our study found that the ACA-PI was positively correlated with the PDI and MDI scores and that the MCA-PI was only positively correlated with the PDI in fetuses with CHDs, suggesting that such a fetal cerebral blood flow redistribution is associated with an increased risk of adverse ND outcomes. Our previous study^5^ also showed that cerebral blood flow perfusion by 3D power Doppler had an association with ND scores in fetuses with CHDs. The frontal region of the brain, which is primarily supplied with blood by the ACA, is believed to be responsible for intellectual and emotional functions, while the MCA supplies blood to areas that play significant roles in motor control and sensory behaviors. The fetal brain-sparing triggered by a hemodynamic adaptation to hypoxia and nutrition/metabolic abnormalities may not guarantee normal neurodevelopment after birth. In a large population-based cohort study, Roza *et al*.^19^ showed that children with fetal ACA redistribution had higher risks of overall problems, emotional reactivity, somatic complaints and attention problems, while those with fetal MCA redistribution had higher risks of somatic complaints.

This study has some limitations. The fact that this was not a longitudinal study may represent the first limitation. All conclusions were based on a single second trimester observation. Limited longitudinal changes in Doppler parameters in CHD fetus^20^ showed that UA-PI increased toward at the end of pregnancy whereas MCA-PI in the third trimester are similar to second trimester measurements, indicating increasing degree of placental impairment. Such placental insufficiency may also contribute neurodevelopmental abnormality. Second, we performed Doppler measurements in the brain hemisphere proximal to the probe and did not examine fetus subgroups according to gender or lateral location. Fetal brain asymmetry may exist; however, we believe that differences in the cerebral flow Doppler parameters caused by such asymmetry are small and not clinically relevant. Third, ND outcomes are influenced by multiple factors; thus, our results can only show associations between cerebral redistributions and neurodevelopment and cannot demonstrate causation.

## Methods

A prospective, cross-sectional study was carried out at the Second Xiangya Hospital of Central South University in China between February 2013 and December 2016. Written informed consent was obtained from all families, and the study was approved by the ethics committees of the Second Xiangya Hospital. All methods were performed in accordance with the relevant guidelines and regulations. Fetuses with postnatally confirmed CHDs were recruited, and the CHDs were subdivided as follows: 1) hypoplastic left heart syndrome (HLHS); 2) left-sided obstructive lesions (LSOLs); 3) right-sided obstructive lesions (RSOLs); and 4) transposition of the great arteries (TGA). Gestation-matched (±1 week), uneventful normal pregnancies were collected as controls. The exclusion criteria included the following: (1) multiple-gestation pregnancies; (2) small size for gestation age, defined as EFW below the 10th percentile for fetuses of the same gestational age; (3) the presence of extracardiac anomalies and cardiac defects other than those listed in the inclusion criteria; (4) identifiable chromosomal abnormalities or syndromes; (5) persistent fetal arrhythmia; and (6) maternal complications, including gestational diabetes, pre-eclampsia and thyroid disease.

Routine obstetrical ultrasound and complete echocardiography were performed on each fetus by one operator (Z.S.) using a Voluson E8 system (GE Healthcare, Milwaukee, WI, USA) with an RAB 4–8-D curvilinear probe. Pregnancy duration was estimated from the day of the last menstrual period and was confirmed by an ultrasound measurement during the first trimester. Fetal biometry was performed, and estimated fetal weight (EFW) was calculated using Hadlock’s formula^21^ and recorded as percentile^22^. Standard and multiple views of each fetal heart were obtained to evaluate the cardiac anatomy. A pulsed Doppler examination of the UA was performed in a free loop of the umbilical cord.

**Figure 4:**
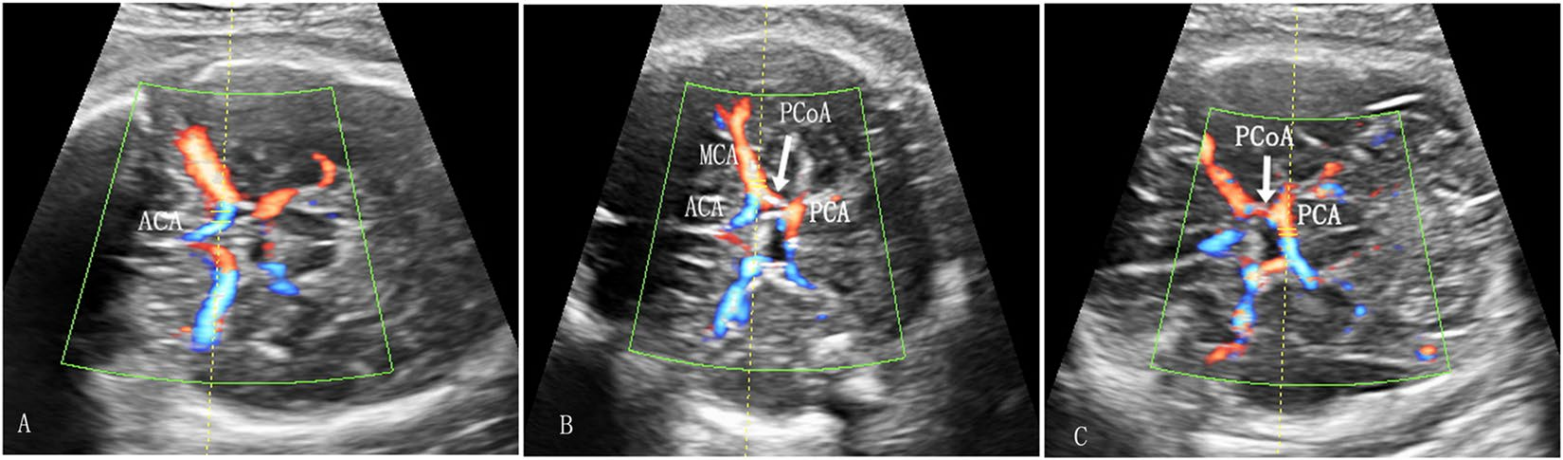
The fetal brain circulation was examined by color Doppler imaging in an axial plane at the level of the circle of Willis. The ACA was recorded with the sample volume placed between its origin from the internal carotid artery and the anterior communicating artery (**A**). The MCA was recorded at the proximal segment after its origin from the circle of Willis (**B**). The PCA was recorded with the sample volume placed between its origin from the basilar artery and the posterior communicating artery (**C**). ACA, anterior cerebral artery; ACoA, anterior communicating artery; MCA, middle cerebral artery; PCA, posterior cerebral artery; PCoA, posterior communicating artery.

Doppler measurements of the three brain arteries were obtained in the absence of fetal movement by the same operator (P.Q.), who was blinded to the group status. Fetal brain circulation was examined by color Doppler imaging in an axial plane at the level of the circle of Willis. The ACA was identified running with a frontal projection and was recorded with the sample volume placed between its origin from the internal carotid artery and the anterior communicating artery. The MCA was identified running anterolateral to the cerebral peduncle and was recorded with the sample volume placed at the proximal segment after its origin from the circle of Willis. The PCA was identified running posterolaterally from the circle of Willis and was recorded with the sample volume placed between its origin from the basilar artery and the posterior communicating artery (Fig. 4). The angle of insonation was maintained below 30°. At least five consecutive regular waveforms were used for an automatic calculation of the PI. For the purpose of this study, the PIs determined from the first examination were used if a fetus was studied on more than one occasion.

The standardized 6-month ND testing was performed on each baby by a single pediatrician (W.T.), who was blinded to the fetal brain hemodynamic and cardiac anatomy data, using the Bayley Scales of Infant Development, Second Edition (BSID-II)^23^. The BSID-II provides two summary scores, the psychomotor development index (PDI) and the mental development index (MDI), with which to assess motor and mental development from 1 to 42 months of age.

The data are reported as the means with standard deviations or frequencies with percentages, as appropriate. The clinical features and ND test scores were compared between the fetuses with CHDs and the controls using Student’s t-tests or χ^2^ tests. Differences in head biometrics and cerebral artery PIs were analyzed by a one-way analysis of variance (ANOVA) with post hoc Games-Howell testing. Signs of fetal cerebral vasodilatation were present when cerebral artery PIs were below the 5th percentile^11,24,25^. Cases with cerebral vasodilatation were recorded and compared in terms of the ACA, MCA and PCA in each CHD subgroup using χ^2^ tests. Correlations between the cerebral artery PI and ND scores were tested using Pearson’s correlation coefficient. To assess interobserver variability, the PIs of the cerebral arteries were independently measured by a third reader (C.D.), who was blinded to the clinical data of 30 CHD fetuses. Intra- and interclass correlation coefficients were estimated in 20 normal cases in the ACA and PCA. P < 0.05 was considered significant. All statistical analyses were performed using PASW statistical software [PASW (SPSS) statistics, version 18.0, IBM].

## Conclusion

There were different Doppler vasodilatation characteristics among the three main cerebral arteries in fetuses with CHDs. The ACA exhibits signs of vasodilatation more frequently and severely than the MCA. The ACA-PI appears to be more sensitive for predicting abnormal ND outcomes than the MCA-PI.
